# Lab to Fab Process Using Ablation Lasers: A Lightweight, Flexible, and Biocompatible Microheater for Wearable Therapy Applications

**DOI:** 10.1021/acsabm.5c01263

**Published:** 2025-10-17

**Authors:** Bhavani Prasad Yalagala, Tahereh Masalehdan, Changhao Ge, Mahmut Talha Kirimi, John Mercer, Morteza Amjadi Kolour, Hadi Heidari

**Affiliations:** † Microelectronics Lab (meLAB), School of Engineering, University of Glasgow, Glasgow G12 8QQ, U.K.; ‡ School of Biomedical Engineering, University of Glasgow, Glasgow G12 8QQ, U.K.; § Institute of Cardiovascular and Medical Sciences/British Heart Foundation, University of Glasgow, Glasgow G12 8QQ, U.K.

**Keywords:** wearables, ablation laser, microheaters, Parylene C, gold electrodes, flexibility, biocompatibility

## Abstract

Wearable thermal therapy has attained significant attention owing to its potential in various wearable and biomedical applications specifically targeted for accelerated wound healing using smart bandages. Here, in the current work, a large area, precision, and scalable fabrication methodology is proposed using an ultraviolet (UV)-based ablation laser as compared to conventional fabrication methods such as photolithography or printing. This laser-based approach is unique and offers rapid prototyping, superior material versatility, design flexibility, and minimal thermal damage to the emerging biocompatible polymer-based flexible substrates. A thin, flexible, and biocompatible microheater and an array (4 × 4) of diverse designs, including circular, hexagonal, and planar, were designed and fabricated on a gold-coated PAC substrate using the proposed ablation laser-based approach. Multiple heater sizes varying from small to extra-large were fabricated and are tailored for the targeted temperatures ranging from 30 °C to 100 °C for biomedical applications, especially wearable wound healing applications. Electrical and electrothermal characteristics revealed that the sheet resistance, thermal response, and response time vary with the structure and size of the microheaters. Further, mechanical flexibility and biocompatibility studies on the PAC, patterned gold electrodes, and polyimide substrate demonstrated excellent mechanical robustness and biocompatibility, clearly demonstrating its efficacy and ability for wearable and implantable applications. Finally, the proposed research paves the pathway for the fabrication of next-generation wearable and implantable biointegrated flexible microheater devices toward advanced thermal therapy solutions.

## Graphical abstract

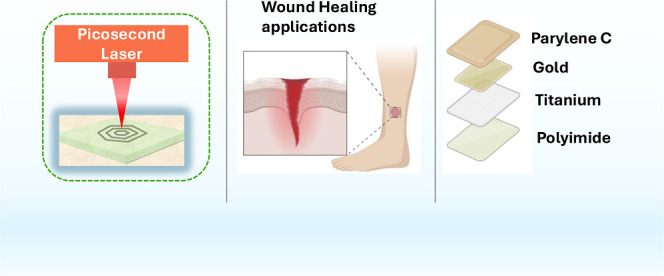

## 1 Introduction

In the evolving area of modern medicine, flexible and conformable microheaters that produce localized and precise heating with a controlled feedback mechanism targeted for a specific tissue region are very crucial.^1–5^ Microheaters have emerged as one of the critical components for various biomedical and wearable applications, including gas sensors, microfluidic chips, DNA amplifications, cell cultures, drug delivery, and wound healing, mainly due to their ability to produce precise, controlled, and localized heating profiles.^6–10^ Despite the wide range of applications, traditional microheaters still lack in spatial resolution and dynamic feedback control to automatically tune the temperature values.^11,12^ Moreover, most of the wearable and biomedical applications, including implantable medical devices, need heaters that are miniaturized, lightweight, flexible, highly conformable, biocompatible, and nontoxic.^13^ There is an urgent need for alternative solutions for the fabrication of these devices without any adverse effects from harmful chemicals. Advanced laser micromachining technology offers these advantages in terms of design flexibility, scalability, precision, material versatility, fast processing speeds, and a contact-free methodology.^14,15^ Further, these laser-based technologies provide excellent repeatability, room temperature etching, lower power consumption, and medium-scale manufacturability and can be operated in a low-cost, cleanroom-free environment, offering an eco-friendly and sustainable methodology for the fabrication of future device technologies.^16^ The advanced and energy-efficient laser technology provides an excellent transformative approach into the realm of biomedical, wearable, and electronic applications.

Despite several advancements in the design and fabrication of the flexible microheaters, there is a need for improvements in terms of stability, repeatability, and precision of the temperatures achieved. A low operating range of temperatures with a heater precision is crucial for biomedical and wearable devices.^17^ These types of devices, by their nature, will damage tissue outside the normal physiological temperature range. This might be required, such as bulk tissue ablation, but finer control would allow a more physiological controlled response.^4^ Highly conducting electrode materials including silver (Ag), platinum (Pt), copper (Cu), gold (Au), and tungsten (W) have been explored as electrode materials for the fabrication of thin film microheaters owing to their excellent electrical, thermal, chemical, and mechanical properties.^18^ Gold is a desirable electrode material due to its excellent electrical conductivity (50 MS/m). Platinum Iridium (90:10) is also favored due to its corrosion resistance, oxidation resistance, ductility, greater mechanical flexibility, and superior biocompatibility as compared to the other metals such as Cu, nickel (Ni), and titanium (Ti). On the other hand, multiple rigid and flexible substrates such as glass, silicon, ceramics, paper, and polyimide have been explored for the fabrication of microheater applications.^19^ However, a substrate with greater conformability, ultralight weight, good biocompatibility, and high thermal stability is ideal for most of the wearable applications. Parylene C (PAC) is a popular and FDA-approved material which exhibits good flexibility, high temperature withstanding capability, lower surface roughness, and biocompatibility.^20^ Further, PAC also offers pinhole-free conformability, low thermal conductivity, superior chemical resistance, and high electrical insulation, making it one of the preferred choices for both encapsulation as well as the substrate material.^21^ Achieving high-temperature power-efficient microheaters is challenging, and a detailed design optimization is crucial to achieve the desired performance. Another important parameter is the fast response and recovery time of microheaters for reaching highly dynamic temperature profiles during their operation for wound healing applications. Recently, Long et al. have demonstrated flexible and low-power microheaters using tin oxide films for gas sensor applications.^22^ Similarly, Lan et al. demonstrated a work on a high-performance, easy-to-fabricate microheater, proposed a cost-effective method for the fabrication of microheaters using a printing technology using a composite material made of silver nanowires and Poly-Vinyl-Alcohol (PVA).^23^ In a similar way, Fan et al. proposed an integrated microheater array with a control loop mechanism for a temperature regulation mechanism using magnetic nanoparticles to operate at microwave frequencies.^24^ The array is fabricated using the spin coating method by mixing the magnetic nanoparticles in the PDMS. Various types of methodologies, including photolithography, spin coating, and more advanced printing technologies (e.g., screen and inkjet printing), are being explored. Although printing technologies are scalable and easy to fabricate, they suffer from device-to-device variability, large surface roughness, adhesion issues with the substrates, and other mechanical stability.^7^

Herein, a unique microfabrication method is presented for the fabrication of flexible and implantable microheaters using UV-based ablation lasers targeted for wearable thermal therapy applications, especially for accelerated wound healing. A systematic study is performed on optimizing different laser parameters such as the laser power, speed, and number of repetitions. Ultrathin, lightweight, biocompatible, and wearable microheaters are manufactured using Cu electrodes and a pinhole-free and transparent PAC substrate. Various designs, such as circular, hexagonal, and planar electrodes of multiscale sizes, are demonstrated. Various electrical measurements, such as sheet resistance, conductivity, and *I*–*V* measurements, are performed to assess the electrical properties of microheaters. To further understand the thermal response of the heaters, electrothermal measurements (DC and pulse) are performed by applying different input voltage and current profiles. The temporal response characteristics reveal that the heat dissipation across microheaters is uniform and quick within a few seconds. Finally, the biocompatibility experiments, mainly the live–dead assay, are conducted using the L929 cells on the as-fabricated microheater design to demonstrate its applicability in wearable and implantable applications.

## 2 Experimental Section

### 2.1 Materials

The PAC was purchased from specialty coating systems during the purchase of the deposition chamber. The microscopic glass slides were purchased from Fisher Scientific UK. An infrared (IR) camera was purchased from the Testo 871 s thermal imaging camera systems. Mouse aorta smooth muscle cells (MASMCs) were isolated previously from mouse aortas of C57BLK6/J mice (Envigo—UK).^25^ A stock of both cells is stored at −135 °C in liquid nitrogen storage within sterile cryogenic storage vials (E3110-6122, StarLab). They are stored at a density of 1 × 10^6^ cells/mL in 10% dimethyl sulfoxide (DMSO) (D/4120/PB08, Fisher scientific, UK). To culture the cells for experiments, the vials were rapidly defrosted in a 37 °C water bath. 1 mL of culture media was added to the thawed cells, and the cells were spun at 1500 rpm for 5 min. The supernatant was removed, and the pellet was broken up in 1 mL of culture media. The suspended cells were then transferred to a vented T75 culture flask (430641, Corning, USA). MASMCs were cultivated in Dulbecco’s Modified Eagle’s Medium (DMEM) (21885025, Gibco, Thermo Fisher) with 10% fetal bovine serum (FBS) (10270106, Gibco, Thermo Fisher) and 5% penicillin (10,000 units) streptomycin (10 mg) (P0781, Sigma-Aldrich, Merck). Culture flasks containing cells were incubated in an incubator at 37 °C with 95% relative humidity and 5% CO_2_. Every experiment that required cell culturing was placed within an incubator with the same temperature, humidity, and CO_2_ levels of 37 °C, 95%, and 5%, respectively.

Initially, the culture medium is aspirated and discarded from the T75 flask. The flasks are then rinsed twice with 5 mL of Dulbecco’s phosphate-buffered saline (PBS) (14190094, Gibco, Thermo Fisher) to eliminate excess calcium. Following the removal of PBS, cells are trypsinized from the flask using 2.5 mL of a Trypsin–EDTA solution (T3924, Sigma-Aldrich, Merck). Subsequently, the cells are returned to the incubator for 5 min and examined under a microscope for detachment from the flask. Once detachment is observed, the cells in Trypsin–EDTA are transferred to a sterile centrifuge tube (430790, Corning, USA). The Trypsin–EDTA is neutralized with 2.5 mL of DMEM with HEPES, and the mixture is aspirated from the tube. A portion of this stock solution is then placed on a hemocytometer for cell counting. The centrifuge tube is spun at 1500 rpm for 5 min, after which the supernatant is discarded, leaving a pellet of cells. The pellets were disaggregated and resuspended in DMEM with HEPES to create a stock solution. Before cell seeding, each experimental material was sterilized with 70% ethanol and rinsed thrice with dH2O to remove any ethanol residue within a sterile laminar flow hood. These experimental materials were then placed in their respective well. Cells were subsequently seeded at 300,000 cells per well in two sterile 6-well plate cell culture plates (Thermo Fisher, Massachusetts, United States) and incubated for 24 h. After 24 h of cell proliferation, Acridine Orange/Propidium Iodide staining solution was prepared to stain SMCs. Acridine orange was prepared by dissolving 5 μL of 10 mg/mL of acridine orange (AO) (Sigma-Aldrich, USA) in 10 mL of PBS first. A 1.5 mL propidium iodide solution (PI, eBioscience, San Diego, United States) was then mixed with AO to cover the cell monolayer in equal amounts, e.g., 50 μL of AO mixed with 50 μL of PI to form the final staining with a volume of 100 μL. Before staining, all previous culture media were removed, and the cell monolayer was washed with PBS to remove any residue. Culture plates were then carried and mounted to an Olympus IX71 (Olympus Corporation, Japan) for fluorescent imaging once the staining solution was pipetted into each well of the culture plates. Before imaging, a specially built heating hood around the microscope was heated to 37 °C to improve the atmospheric conditions for SMCs during imaging.

### 2.2 Preparation of Parylene C Film

A large, microscopic glass slide with dimensions of 7.5 cm (length) and 5 cm (width) was used as a reference substrate for the growth of the thin layer of PAC film. Initially, the glass slide is cleaned with acetone, methanol, and 2-propanol solutions in an ultrasonication bath for 10 min. The cleaned glass slides were loaded into the vertical stacks of the SCS Parylene C coating machine. The Parylene C dimer powder of 100 g (approximately) was loaded into the process line with a small holder tube connected to the chemical vapor deposition (CVD) chamber. The vacuum pump and chiller are connected to the chamber, and the deposition is carried out at a pressure of 10–50 mTorr and at a temperature of 700 °C. The process is mainly divided into 3 steps, namely, thermal vaporization, pyrolysis, and polymerization. During thermal vaporization, the dimer is slowly converted to the gaseous state after it gets heated to the temperature of 200 °C, and then the vapor spreads inside the pyrolysis chamber, and a thermal decomposition occurs at a temperature range between 650 °C and 700 °C to form a multiple monomer unit by splitting the dimers of PAC. The monomers enter the CVD chamber to form a more uniform and conformal coating on the substrates spontaneously, and the chamber is maintained at room temperature at a very low pressure.

### 2.3 Microfabrication of the Microheaters Using a Picosecond Laser

The flexible microheaters were prepared by using the highly conducting gold thin film-based electrodes. A thin layer of Ti/Au of thickness 10/90 nm was used as the electrode layer and is deposited using electron beam evaporation at an ultrahigh vacuum pressure of 2 × 10^–6^ mbar inside the class 1000 cleanroom in the James Watt Nanofabrication center (JWNC). The previously prepared CVD-grown PAC with a thickness of 50 μm was used as a flexible substrate for gold deposition. Next, an ablation laser from the LPKF system (Germany) emitting a laser wavelength of 335 nm is used to pattern the thin film of gold layer and, as prepared, the gold-coated PAC film. The etching of the gold layer is processed at room temperature without causing any adverse effect on the substrate, and a precise etching of a few nanometers is possible with ultralow power and moderate speeds, with a smaller number of repetitions. Computer-aided (CAD) software Adobe Illustrator and SolidWorks are used to design circular, hexagonal, and planar heater designs of various sizes, varying from small to extra-large. Similarly, various optimization studies, such as power, speed, and number of repetitions, were optimized using the dedicated control software from the LPKF systems. Finally, large-area and flexible microheaters of various proposed designs were successfully fabricated and were finally encapsulated using PAC to protect them from external environments.

### 2.4 Electrothermal Characteristics of Microheaters

The current vs voltage measurements were performed using a manual 4-point probe station connected with the Agilent source meter (model no B2912) with an Easy Expert software connected to it. Similarly, the power and heating measurements were performed using the externally connected DC regulated power supply from RS components with a maximum voltage and current values of 30 V and 2A, respectively. The sheet resistance and conductivity measurements were performed using the 4-point probe measurement system from Ossila instruments, with a *R*_s_ range varying from 100 mΩ/sq./sq. to 10 MΩ/sq. with an equal gap in between the probes of 1.27 mm. The thermal profiles and temperature measurements were captured using an infrared (IR) camera and were recorded using the associated software application.

### 2.5 Thermal Response under Electrical Pulse Stimulations

Pulse experiments were conducted on the microheaters to evaluate the response speed and heating capacity of the microheaters. Pulse control was programmed using an Arduino microcontroller. As shown in Figure 5, the microheater was connected to a current source with a MOSFET employed as a switch. When the Arduino output was set to high, the switch turned on, and when the output was set to low, the switch turned off. Temperature measurements were obtained using an NTC 10 Kω 3950 thermistor module. This type of sensor adheres to the following relationship between temperature and resistance:^26^

$$R=R_{0}\cdot e^{\beta \cdot \left(\frac{1}{T}-\frac{1}{T_{0}}\right)}$$

where *T*_0_ is the reference temperature, *R*_0_ is the reference resistance at the reference temperature, *β* is the temperature sensitivity coefficient, and *e* is the base of the natural logarithm. In this sensor, *T*_0_ is 25 °C, *R*_0_ is 10 Kω, and *β* is 3950. The temperature can be obtained by a mathematical calculation from the measured sensor resistance, where *T*_0_ is the reference temperature (25 °C), *R*_0_ is the reference resistance at *T*_0_ (10 Kω), *β* is the temperature sensitivity coefficient (3950), and *e* is the base of the natural logarithm. The temperature was derived mathematically from the measured sensor resistance. The resistance of the sensor was determined by measuring the voltage across a voltage divider circuit, which consisted of a 10 Kω resistor connected in series with the sensor. In summary, the Arduino calculates the resistance by acquiring the divided voltage through an analogue input and subsequently computes the temperature based on the measured resistance. The data were updated every 2 s and transmitted to a computer via serial communication for recording and analysis. Based on this system, two pulse experiments were conducted. Single Pulse Experiment: In this experiment, the power was turned on once to raise the heater’s temperature from room temperature to a steady state. The power was then turned off, allowing the heater to cool naturally to room temperature, while temperature changes were recorded. Cyclic Pulse Experiment: In this experiment, the current was repeatedly turned on and off at a fixed interval (300 s), causing the heater to undergo multiple heating and cooling cycles. Temperature changes were recorded throughout the process.

### 2.6 Simulation Study Using COMSOL and Bending Studies

COMSOL simulation can predict the heat distribution of the heater and provide a reliable basis for the design optimization of the heater. The electrical and thermal coupling time-dependent simulation of the microheater was conducted using COMSOL 6.2 Multiphysics. The simulation focused on analyzing the heat generation within by electrical current flow. Three designs were modeled as the L level size and compared to assess their temperature distribution when subjected to a 1 V source (DC). Semicircular-shaped modules of different radii varying from 30° to 180° were designed using the CAD software SolidWorks and were printed using a simple 3D printer. The as-fabricated microheater designs of different shapes were molded on top of the 3D modules, and the *I*–*V* measurements were performed using a 4-point manual probe station connected with the source meter from Agilent systems. All the measurements were performed at ambient temperatures, and the probe station is enclosed in a Faraday cage to prevent external measurements.

## 3 Results and Discussion

### 3.1 Design of Various Flexible Microheaters

Flexible microheaters using thin films of metal electrodes are two of the potential applications of the personalized and user-friendly wearable thermotherapy and wound healing, providing localized heating with very precise control of temperature. Figure 1a illustrates the schematic representation of the concept of a smart bandage with microheaters, demonstrating the application of the microheaters and their potential use for the speedy recovery of the wound. These microheaters can be very helpful in multiple ways for accelerated wound healing by promoting increased blood circulation, resulting in cell proliferation, helping in moisture control, helping in the prevention of bacterial infections, and enhancing the formation of cell growth. Here, for this work, three design shapes of the microheaters, such as circular coils, hexagonal coils, and planar coils, were designed using the AutoCAD software. Further, based on the size, each shape has four different types of sizes and is named short (S), medium (M), large (L), and extra-large (XL), as shown in Figure 1b. A large area PAC is used as the substrate of thickness 50 μm prepared using the standard PAC deposition coater. Next, the thin film of titanium/gold (Ti/Au) of thickness 10 nm/200 nm was used as the electrode material and was uniformly deposited using the E-beam evaporation. An advanced pulsed ablation-based picosecond laser is used for the etching of the top conducting layers (Ti/Au) on the PAC film without any adverse effect on the substrate.

**Figure 1:**
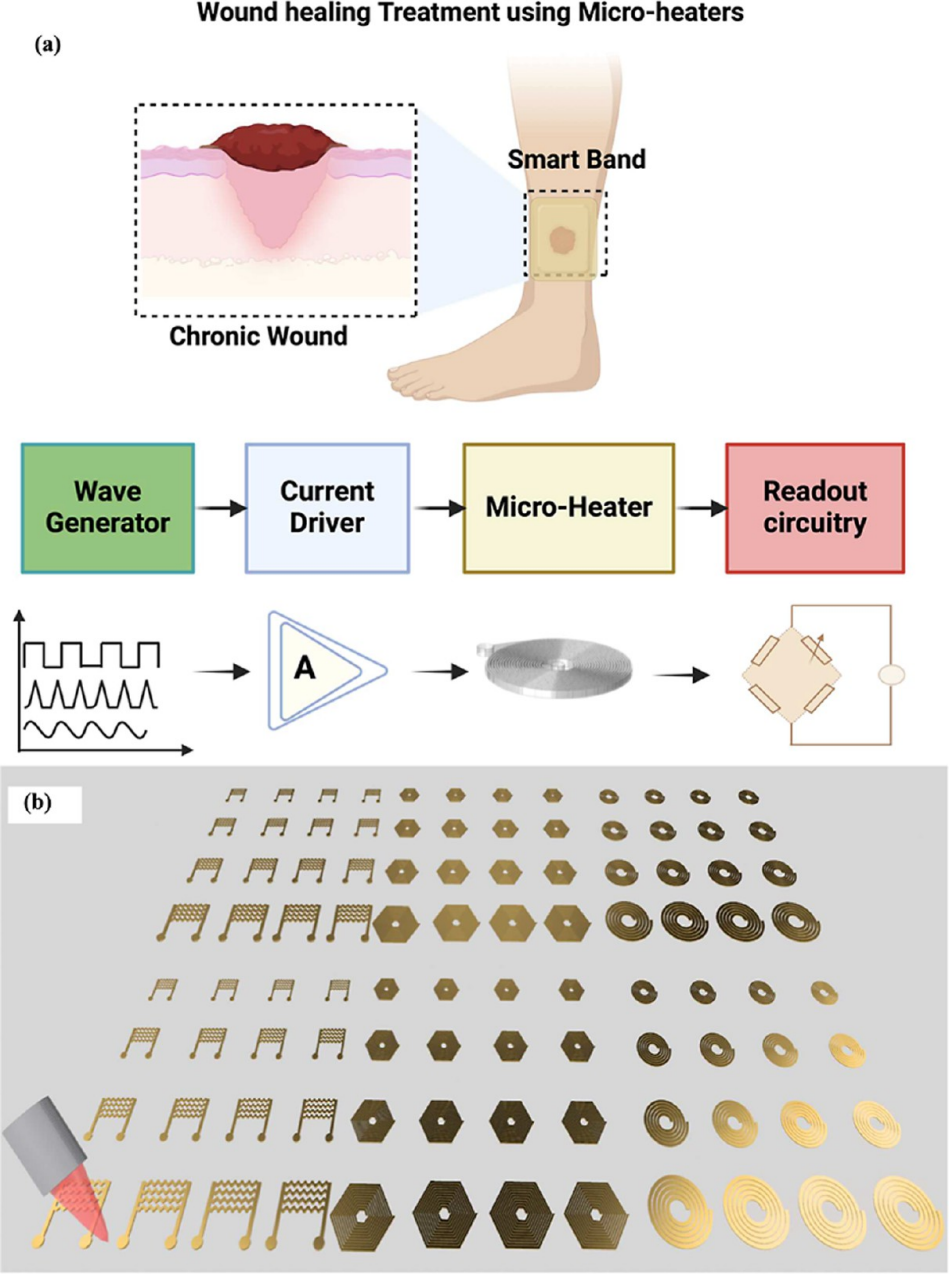
(a) Conceptual illustration of the use of the wearable and biocompatible microheaters embedded in a smart bandage for wearable thermal therapy especially targeted toward accelerated chronic wound healing and associated readout circuitry including the waveform generators, amplifiers, and other readout circuitry for the controlled generation of the heat (top) and (b) schematic representation of the fabrication strategy proposed for the fabrication of the flexible microheaters using an advanced UV-based picosecond laser providing small-scale manufacturability and design flexibility.

### 3.2 Fabrication Using Ablation-Based Pulsed Lasers

The ablation pulsed laser methodology proposed significantly helps in achieving the precise patterning by the layer-by-layer etching, typically a few nm on a wide variety of materials, especially thin films of metals, semiconductors, and dielectrics. Moreover, it can also provide the advantage of cutting and thinning the microfilms and slightly thicker substrates as well, with minimal damage to the substrate. Further, the lasers can offer advantages in terms of design flexibility and a completely cleanroom-free fabrication process, providing material versatility, especially thermally sensitive biodegradable and biocompatible polymers. Fine-tuning the properties of the laser parameters, such as laser power, frequency, and number of repetitions, is crucial to achieve the desired performance of the microheaters with greater precision. From the microscopic observation, it is spotted that the use of excessive laser power (>1 W) at a speed of 180 mm/s for 2 repetitions can cause damage to the sample due to the overheating, leading to the microcracks, burns, and delamination in the polyimide film of thickness 100 mm as shown in the Supporting Information in Figure S1. Lower powers like 100 and 500 mW at the same scanning speed and repetition as mentioned result in the incomplete etching of the gold layer with slight residues of the gold hatching layers observed, resulting in a conductive channel interconnectivity confirmed using a simple multimeter check. An imperfect structuring between the lines further resulted in the abnormal distribution of the heating, affecting the final performance of the microheater, with the low- and high-magnification images shown in Supporting Information Figure S1a–d. The lateral and overlay resolutions are predefined to 5 and 2 mm, respectively, and the cross-selection pattern was selected for etching of the metallic layers. Next, a moderate power of 1000 mW at a slightly lower scanning speed of 120 mm/s for 2 repetitions was used to obtain perfect etching for all the proposed designs, such as circular, hexagonal, and planar, as shown in Figure S1e,f (low and high magnifications). The ultrashort pulses offer room temperature etching without affecting the intrinsic properties of the substrate, owing to very short life span interval times of the laser, providing lower thermal diffusions, generating a negligible heat-affected zone associated with the laser beam. Interestingly, it is observed that the increase in the power and number of repetitions results in higher etch rates, and it is preferable to choose the moderate power with increased repetitions to avoid any potential damage to the substrate, as demonstrated in Supporting Information Figure S1g,h. Finally, the complete gold layer was etched off after the hatching power was increased further to 1500 mW, which might be due to the excessive heat generation creating a rapid vaporization instead of the ablation by overcoming the thermal diffusion as shown in Supporting Information Figure S1i,j. Here, one primary reason for selecting the UV laser of wavelength (335 nm) is due to its precise layer-by-layer etching of a controlled depth of 10–20 nm with minimal thermal damage owing to the short absorption depth. This UV laser often offers higher photonic energy, crucial to achieve very low etch rates, facilitating the strong photonic interaction with the material surface. These UV lasers also provide superior precision to help achieve a clean and particle/residue-free micropatterning of various metals, including Au, Ag, and Ti. Here, the Ti/Au-based electrodes are well-known for their stability and chemical inertness, along with noncorrosive and nonoxidative behaviors. Also, to avoid overetching of the surface of polyimide, the laser parameters were fully optimized by precisely controlling the hatch power of the laser. Further, to better understand the change in the surface properties of the flexible polyimide, a high-resolution microscopic image is captured, showing a minor or non-noticeable variation, as shown in Figure S2a–c on the surface. Next, the material properties on the surface of the microheater before and after laser treatment were studied using XRD and FTIR characteristics, and the obtained results are presented in the Supporting Information, Figure S2d,e. Both the XRD and FTIR results reveal that no additional peaks were observed related to carbon or graphene. A Raman spectroscopy study was conducted on both laser-etched and unetched polyimide film, but there were no proper spectral peaks with very weak intensities, and a spurious line was exhibited with no characteristic bands. Table S1 shows the full list of laser parameters, including isolation power, number of repetitions, and scanning speed, for the fabrication of the microheaters. Multiple parameters like the number of turns (*N*), metal thickness (*t*), spacing (*d*), and size (*s*) will impact the performance of the heater, and the designs proposed are represented in Figure 2. Figure 2a,c,e shows the schematic designs of different sizes, such as small to extra-large, of the proposed circular, hexagonal, and planar coils using CAD design tools, and the same files were used for the simulation studies and for the fabrication using lasers. Here, to make the design uniform *t*, *N*, and *d* were kept as uniform and have considered the influence of design and size on the thermal response. A more detailed discussion on the electrothermal response characteristics is presented in the section below. Figure 2b shows the multiple sizes of the circular coils, i.e., S, M, L, and XL, ranging from 1 to 3 cm in 4 different rows with uniform spacing on a smooth and thin PAC substrate. The fabricated coils are very smooth and uniform and are perfectly etched without any gold particles left after the etching, demonstrating the clean, precise, and contact-free repeatable fabrication process very useful for the small-scale batch production of these types of devices. More importantly, the edges are very sharp without much deviation in the proposed shapes, further suggesting their effectiveness in terms of the fabrication. A similar methodology is adopted for the fabrication of the other proposed designs, such as hexagonal and planar heater devices, as shown in Figure 2d,f, respectively. The microscopic images shows that the laser-based fabrication is repeatable without much deviation from the proposed theoretical design. Furthermore,, the processing times are very low in less and it is easy to prepare 10^3^ devices in approximately 30 min (considering 30 s for 16 devices), and the approach is large area compatible with an approximate work area of 210 mm × 297 mm (nearly A4 size) and can be finished in one batch.

**Figure 2:**
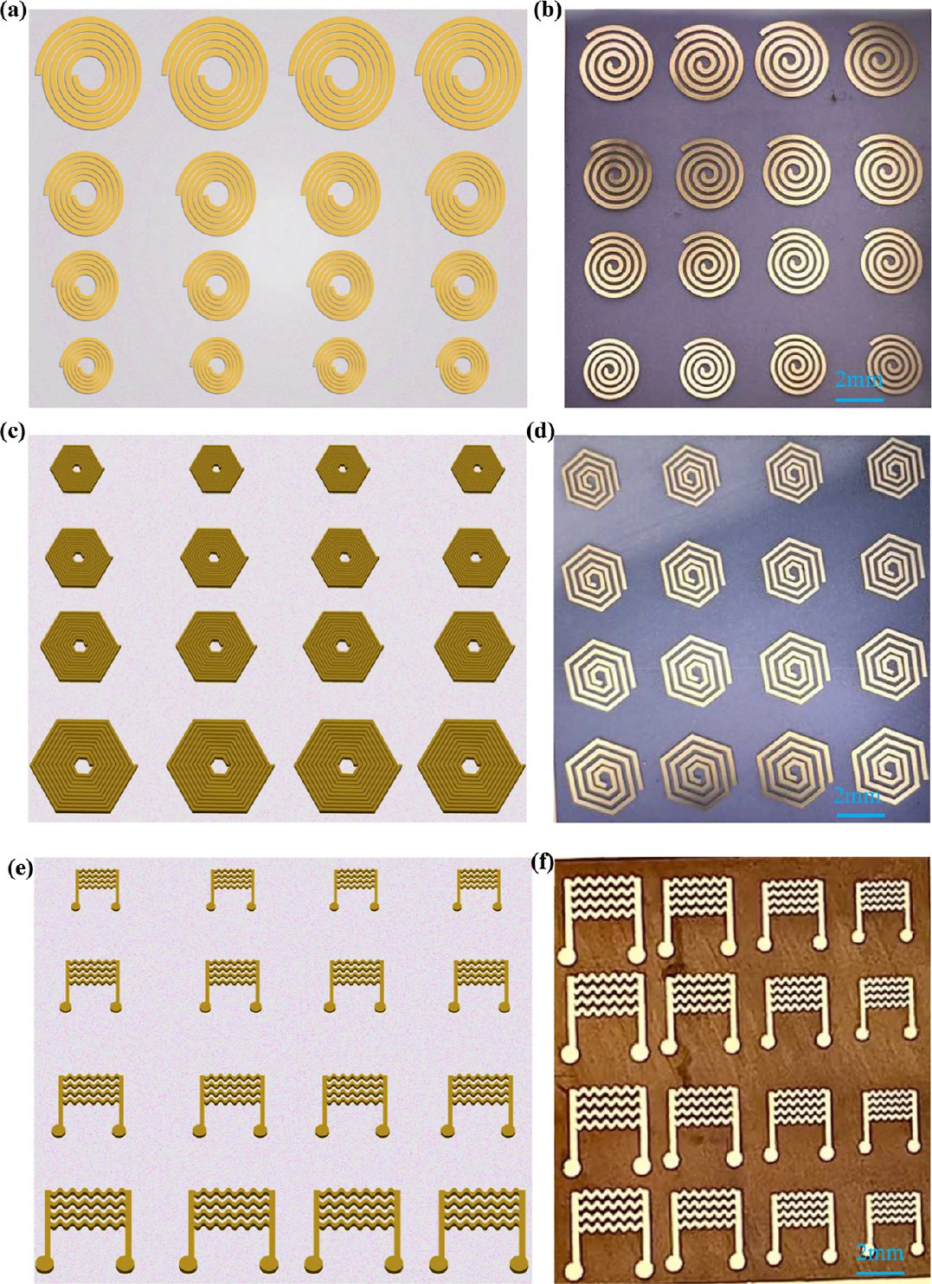
(a–f) Schematic representation of the array (4 × 4) of the proposed microheater designs (left) of multiple sizes varying from small (S) to extra-large (XL) and the microscopic images of the fabricated microheaters (right) using a high-speed UV-based picosecond laser representing its preciseness and accuracy on multiple flexible substrates (Parylene C and polyimide) without effecting the inherent properties of the substrates.

### 3.3 Electrical and Thermal Characterization

Electrothermal characterization is very crucial for evaluating the performance of the flexible microheaters to ensure precise and uniform heating around the area. Initially, to test the electrical characteristics of the device, current (*I*) versus voltage (*V*) measurements were performed on the laser-fabricated microheaters. Figure 3a–c shows the absolute *I*–*V* characteristics of the circular, hexagonal, and planar electrodes of different sizes for an applied voltage range of 0 V to +3 V for a compliance current of 100 mA. The compliance current is set to protect the microheaters from excessive overheating and protect the devices from permanent damage. As expected, all the microheaters have demonstrated excellent linear/ohmic behavior owing to the excellent conductivity of the gold electrodes. Moreover, the results confirmed that there are no major deviations in terms of the fabrication process, and there are no microcracks or breaks owing to the heat transferred onto the substrate. Achieving a smooth linear curve is very crucial for uniform heat distribution and is observed for all the fabricated devices, demonstrating the repeatability of the fabrication process. A more magnified version of the device behavior is noticeably represented in the inset of each Figure 3a–c, showing the minor difference in terms of the magnitudes of the current values. The results suggest that the circular and planar coils are highly conducting as compared to the hexagonal electrodes, which might be due to the reduced sharp corners as compared to the 120° angle of the hexagonal, thereby clearly inducing a reduction in the overall electron scattering, producing a higher current path efficiency. A more detailed analysis to confirm the hypothesis is clearly discussed in the simulation studies in Section 3.5 of the paper. It is observed that there is a trade-off between the area and the device resistance, and an increase in the size can significantly decrease the resistance of the microheaters. Sheet resistance (*R*_s_) and conductivity (σ) are two of the important properties of the thin film-based microheaters, since these can directly affect the power or thermal dissipation. Theoretically, the sheet resistance can be calculated using eq 1 as represented below.^27,28^

1 $$R_{s}=R\left(W/L\right)=\left(\frac{1}{G}\right)\left(W/L\right)=\frac{r}{t}\left(W/L\right)$$

where *R* is the resistance of the fabricated microheater, *G* is the conductance, ρ is the electrical resistivity, and *W* and *L* are the geometrical dimensions, like width and length of the microheater. The *R*_s_ mainly depend on the geometrical dimensions and thickness of the film. To further investigate the *R*_s_ and σ of the laser-fabricated microheaters, four-point probe sheet resistance measurements were performed. To achieve the desired electrothermal performance, the flexible microheater should have a lower *R*_s_ and higher σ values. The plot of the *R*_s_ and σ for different sizes of the Au-based microheaters is represented in Figure 3d,e, respectively. The obtained results suggest that the *R*_s_ values are typically in the range of mΩ/sq and are inversely proportional to the size of the heaters. The larger circular (XL) design offers the highest conductance of 45 S/m value and the minimum *R*_s_ of <1 mΩ/sq. Further, it is observed that *R*_s_ and σ perfectly coincide with the linear behavior of the samples. For better understanding purposes, a more detailed comparison of the conductivity of the different fabricated microheaters is shown in Figure 3f, and the results show that the excellent conductivity of the microheaters with a very low value of *R*_s_ can be the best microheaters for various implantable and wearable applications.

**Figure 3:**
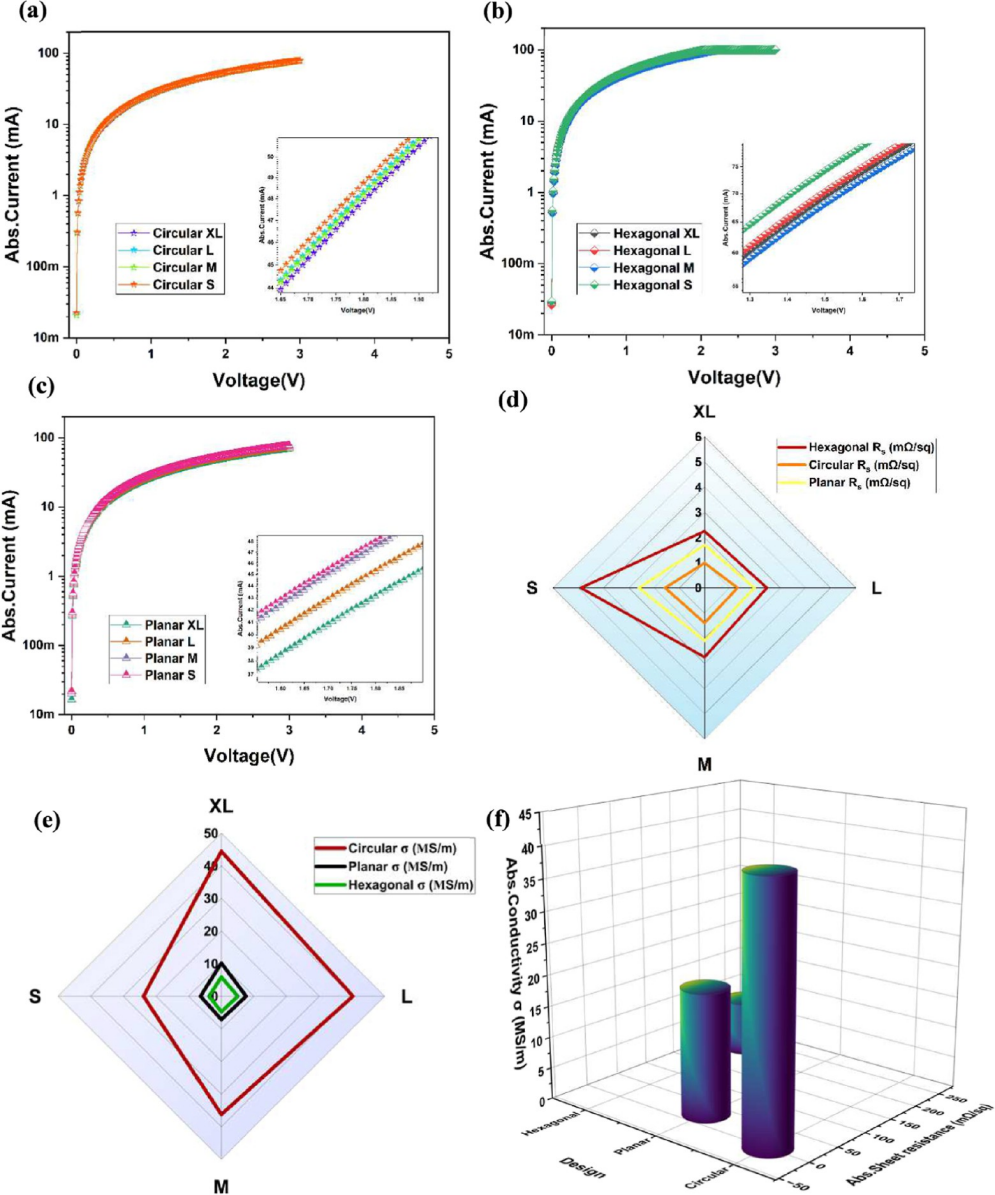
(a–c) Absolute current (*I*) vs voltage (*V*) characteristics of the fabricated circular, hexagonal, and planar designs of the microheaters, respectively, in the positive voltage regions and a magnified view of the variation in the current with increase in the sizes for each design is shown in the insets. (d–f) The comparison of the different electrical performance metrics, like sheet resistance (*R*_s_), conductivity (σ), and the combination of both *R*_s_ and σ.

Wearable thermal therapy is gaining significant attention which requires an array of flexible microheaters that can produce a programmed or controlled thermal profile with greater precision. Especially, these wearable microheaters have potential for many medical and implantable applications such as drug delivery, pain relief, wound healing, and relaxation of muscles.^29–31^ Generally, wearable devices are very much limited in terms of power as it was operated using microbatteries, and a more efficient design that can provide a good thermal response with minimum power consumption is very crucial. Electrothermal characterization is one of the important studies to understand the efficient thermal response with optimal power consumption. The input voltage is applied from the external variable power supply, a probe station is used for the characterization, and the thermal response is measured using the IR camera. Figure 4a,b shows the IR camera image with an inset of the schematic design of the circular coil for a desired mild temperature range of 20–50 °C used for the treatment and 3D plot of the thermal response of the circular microheater of multiple sizes (S, M, L, and XL) under different voltages and powers of 9 V and 3 W, respectively. The results demonstrate that different sizes of the circular coils exhibit a linear response with an increase in temperature with respect to the applied input power (*P*). Moreover, these fabricated large circular microheaters have generated the highest temperature of 95 °C at an input voltage, power of 9 V and 3 W, respectively. The high performance of the microheaters is primarily due to the potential transduction of electrical energy to thermal energy, like a resistor. Although the microheaters can produce more heat of greater than 100 °C at slightly higher powers, it is well-known that the higher thermal values applications might affect the mechanoreceptors in the skin and might permanently damage the cells. From the literature, it is observed that the temperature range for destroying the bacterial infection is below 70 °C. The linear increase is primarily due to the well-known phenomenon, namely, the Joule effect (*P* = *V* ^2^/*R*), i.e., the heat dissipation linearly increases with the applied input power as the material remains unchanged. It is to be noted that the *P* is calculated by using the standard equation of *I* ^2^ *R*, where *R* is the initial resistance of the fabricated coil, considering the device as a linear device. Furthermore, it is observed that an increase in the size of the coil, i.e., S, M, L, and XL, indicates an increase in the heat dissipation or temperature generated due to the change in the overall length of the coil, increasing the resistance of the coil. Similar experiments were performed on the hexagonal design, and the thermal profile images were captured using an IR camera with a schematic design in the inset, and its variation in the thermal response of the different sizes of the coils under different voltages and power was performed on the hexagonal as shown in Figure 4c,d, respectively. Next, the schematics of the planar microheater, IR camera image (inset), and the thermal response characteristics are represented in Figure 4e,f, respectively. An identical trend is observed in terms of both size and input power in both planar (XL) and hexagonal (XL) and has demonstrated a maximum temperature of 100 °C and 80 °C, respectively, at the same 3 W power. All these fabricated Ti/Au microheaters exhibit similar characteristics with identical trends, with a slight change in the temperature values. The electrical to thermal conversion efficiency (η) is one of the crucial parameters to determine the performance of as-fabricated miniheaters, and the results are shown in Supporting Information Table S2a–c for various designs and their respective sizes. The heating efficiency (η) is calculated as the ratio of the change in output temperature (Δ*T*) to the applied input power (*P*). The tabulated results demonstrate that all of the designs and sizes (S, M, L, and XL) have exhibited different efficiencies for the increase in the power values. For circular designs, S has exhibited a higher η, and XL has exhibited a lower η as compared to the other sizes due to the variation in the surface area. In contrast, hexagonal microheaters have demonstrated a higher efficiency for XL size, and planar microheaters have demonstrated a mixed response with a higher efficiency for the large and medium, intensifying a strong temperature-dependent response. These results clearly highlight the significance of the coil shapes and sizes can severely affect the conversion efficiency, and here, it is observed that the small-sized samples can produce relatively larger efficiencies as compared to the larger ones. Planar structures have offered more stable and superior performance with a lower input power.

**Figure 4:**
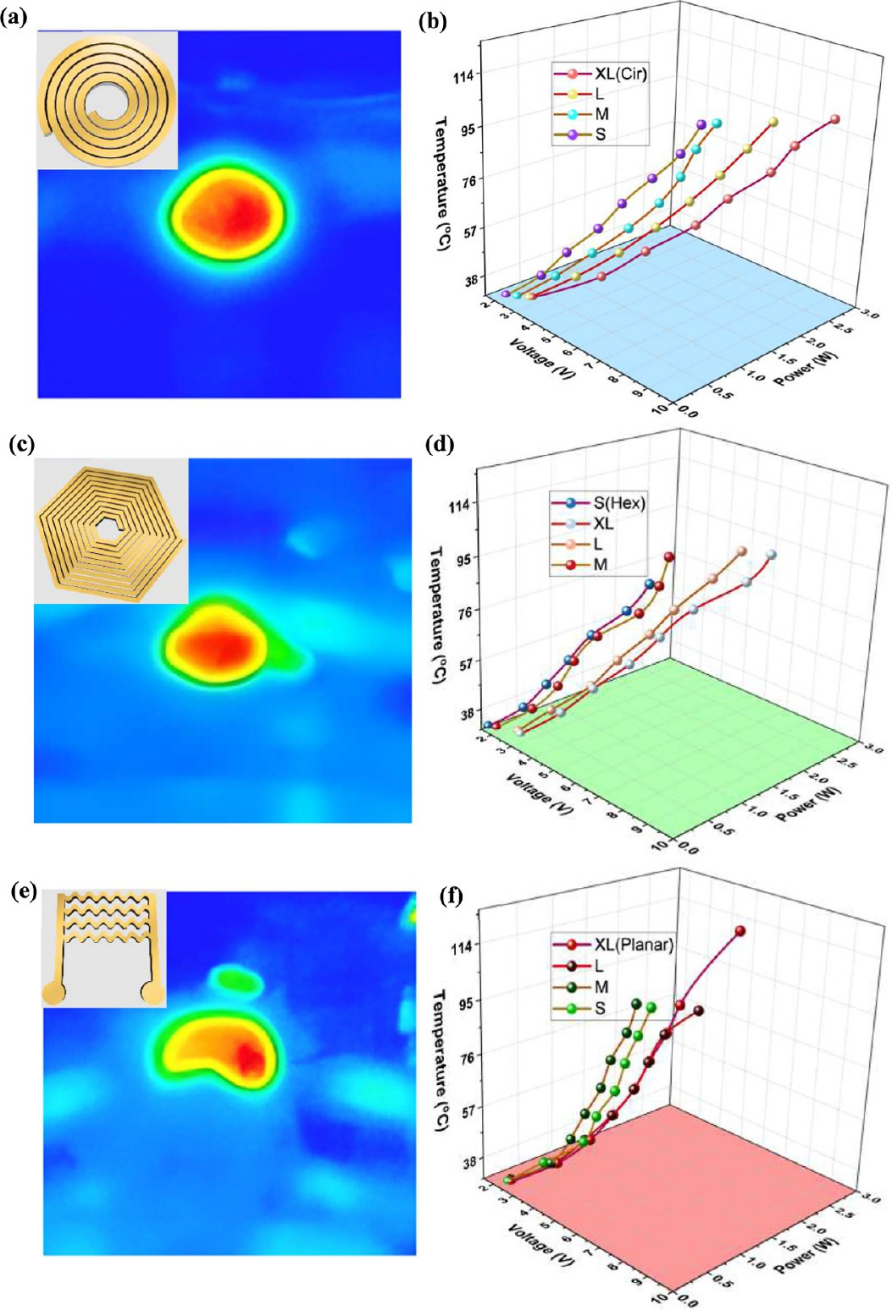
(a–f) Thermal profiles of the multiple designs like circular, hexagonal, and planar with its temperature range varying from 20 to 50 °C and its uniform heat distribution (left) and its corresponding temperature values under different excitation voltages and powers for various sizes (S, M, L, and XL) of each design.

### 3.4 Thermal Response of Microheaters under Pulse Excitations

The single switch experiment reveals the thermal response characteristics, thermal stability, and thermal dissipation properties of the microheater, providing critical insights for optimizing the heater’s design and enhancing its performance. Figure 5a shows the schematic representation of the experimental setup designed for the pulse measurements performed on the fabricated microheaters. The photograph of the actual experimental setup for the pulse measurements by connecting a thermal sensor integrated with the probe tips and the coils is shown in Supporting Information Figure S4a. Next, the proposed flowchart and its control algorithmic flow demonstrating the feedback mechanism used to control the response from the microheaters by regulating the input power to the microheater are depicted in Supporting Information Figure S4b. In this experiment, the microheater was triggered by applying power, demonstrating the change in the temperature, i.e., gradual increase from room temperature (24 °C ± 1 °C) until it reached a stable state (circular: 36.9 °C, hexagonal: 36.9 °C, planar: 37.3 °C), and then the power was turned off and naturally cooled to room temperature. The power was then turned off, allowing the heater to cool naturally to room temperature. While all three designs reached a temperature of around 37 °C in less than 40 s, as shown in Figure 5b, the planar design exhibited 5 more seconds of temperature rising and insufficient stability, fluctuating by approximately 0.2 °C around 37.1 °C. In addition, hexagonal showed the sharpest heating rate, followed by circular, and planar had the slowest heating process. Furthermore, the hexagonal design demonstrated the sharpest temperature rise curve, followed by the circular design, while the planar design exhibited the slowest heating process. However, the planar design cooled faster than the other two after the power was turned off. The cyclic pulse experiment simulates a practical scenario of healing mode, allowing for the assessment of the heater’s stability under repeated thermal expansion and contraction. This provides valuable insights into its reliability, and the results are presented in Figure 5c. While the performance of the three designs is relatively stable overall, there are notable differences in their behavior. Here, the rate of change in the temperature from *T*_0_ to *T*_1_ is defined as the ratio of the difference in the temperature at *T*_1_ and *T*_0_ to the temperature at *T*_0_. Given the periodic nature of this experiment, particular attention was paid to the temperature change rate over a time interval of period *t*_0_, especially the relationship between the two highest temperature points in two adjacent cycles. For circular and planar designs, the temperature extremes fluctuated within a small range. The average periodic change rate of the maximum temperature for the circular design was approximately 0.39%, while the temperature change rate between the maximum temperatures of the last cycle and the first cycle was 3.1%. For the planar design, the corresponding values were an average of 0.27% and a total of 2.2%. In contrast, the hexagonal design exhibited significantly higher maximum temperature variations, with an average change rate of 2.8% and a total change rate of 16.9%. These results indicate that the hexagonal design demonstrates slightly reduced stability in multicycle tasks compared to the other two designs, with a risk of heat accumulation. Between the remaining two designs, the planar design shows slightly better stability than the circular design when considering only the stability perspective.

**Figure 5:**
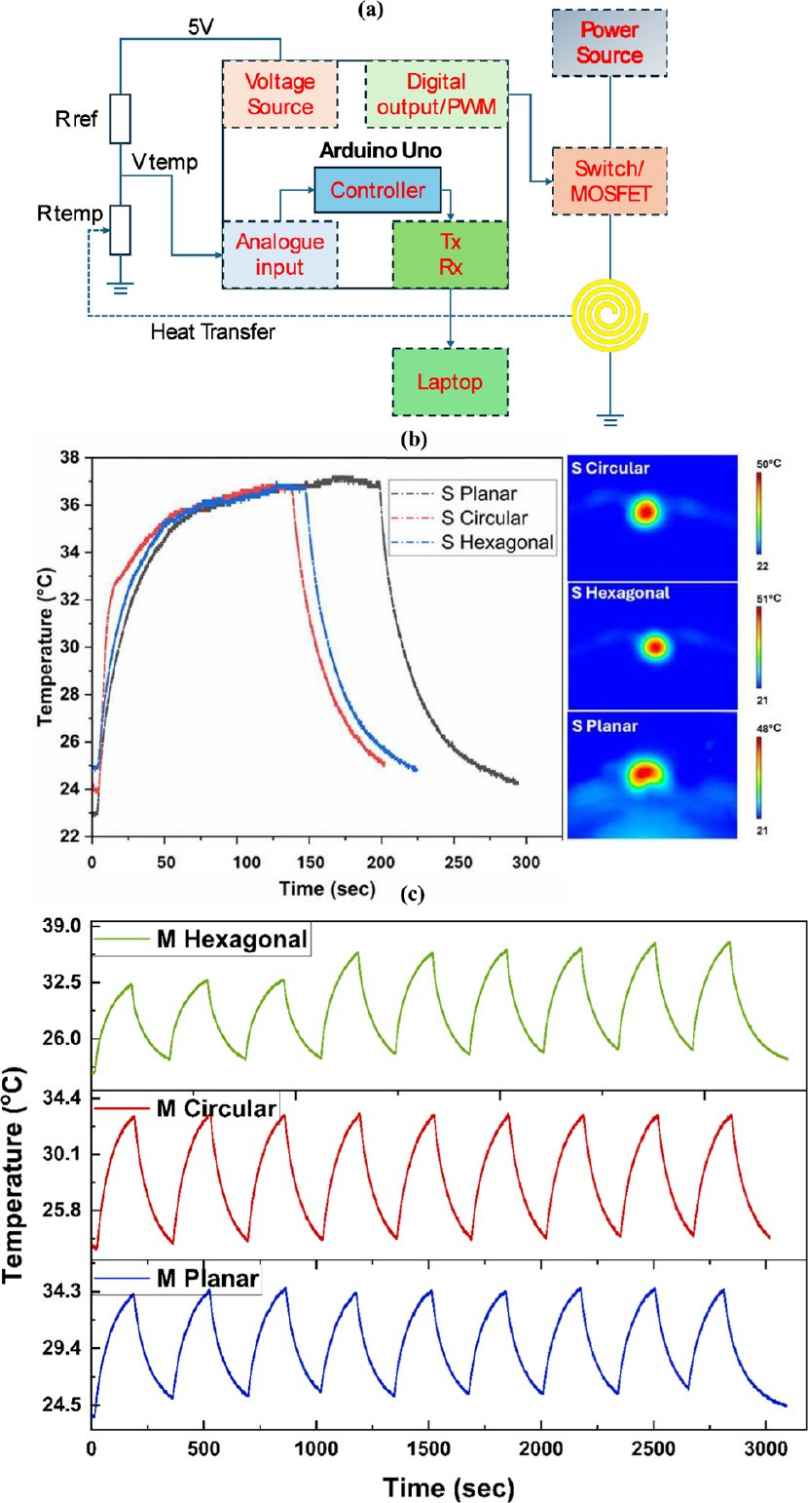
(a) Schematic representation experimental setup designed for the pulse measurements performed on the fabricated microheaters. (b) Thermal response profiles of the different design of the microheaters fabricated using the laser by applying the single pulse of constant amplitude and frequency generated using a pulse source meter and its corresponding heat distribution profiles (right) for various designs to observe the response and recovery speed of the heaters and (c) cyclic stability measurements by the application of the multiple continuous pulses and its response characteristics.

### 3.5 Mechanical Flexibility Studies and Simulation Studies

The mechanical flexibility studies are crucial to understand the conformability and reliability of the microheaters as these devices need to conform to the vibrant and irregular surfaces on the human body. Further, these devices are targeted for various biomedical and wearable applications, like a smart bandage for wound healing comprises a lot of abnormal body movements and deformations on the devices.^32^ To test the mechanical robustness of the Au-based microheaters, the device’s electrical response under bending conditions current multiple bending angles. Figure 6a represents the optical image of the as-fabricated hexagonal heater mounted on the surface of the 3D-printed block with a bending radius of 150°. The *I*–*V* measurements were performed on all the microheaters of diverse designs and sizes, and the electrical response characteristic curves of the circular design of small size under different bending radius varying from 0 to 180° in steps of 30° for a voltage range of −3 V to +5 V, as displayed in Figure 6b. To determine the effect of bending radius on the microheater, the percentage change in the resistance is calculated as the performance deviation ratio of the difference between the resistance value under flat and the maximum bending radius, with respect to the flat value of resistance. The maximum change in the response is obtained as approximately 6% calculated using the formulas for the circular design of small size. Similarly, *I*–*V* measurements were performed on the hexagonal and planar device of the same small size for the same voltage range of −3 V to 5 V under similar conditions, with multiple bends, as illustrated in Figure 6c,d, respectively. The obtained results suggest that maximum performance deviation ratios of 66% and 38% are observed for hexagonal and planar microheaters. One possible reason for the excellent power efficiency of the circular coils is the higher conductivity, and these coils can also produce a more uniform distribution of the heat in a radial directiions. The high and low magnification optical images of the *I*–*V* characteristic measurements using the probe stations of the circular microheaters demonstrating the mechanical flexibility under the bending angle of 70° on a 3D printed module are shown in the Supporting Information in Figure S3a,b. Also, the *I*–*V* measurements were performed on the other sizes, like medium, large, and extra-large microheaters of all designs, as is shown in Supporting Information Figure S5. Finally, it is observed that a similar trend has been followed with other design sizes and among all the designs, circular coils have demonstrated a good mechanical robustness with a minor variation in the change in the resistance of the microheaters, directly impacting the heat dissipation. To further evaluate the behavior of the microheaters in a liquid medium, electrochemical impedance spectroscopy measurements were performed on the laser-based microheaters. A neutral ion-free medium mimicking human body fluid is used to understand the variation in the impedance of the microheaters. Here, a PBS solution of a pH value of 7.4 is chosen to perfectly emulate the body fluids, and the EIS plots are shown in Supporting Information Figure S6a,b. The obtained results from the EIS study suggest that the microheaters exhibit an impedance of a few MΩ @1 kHz frequency and confirm that there are no induced defects after the laser etching/ablation was performed. Also, these results suggest that microheaters show a robust resistance to ion adsorption, corrosion resistance, and performance degradation over time.

**Figure 6:**
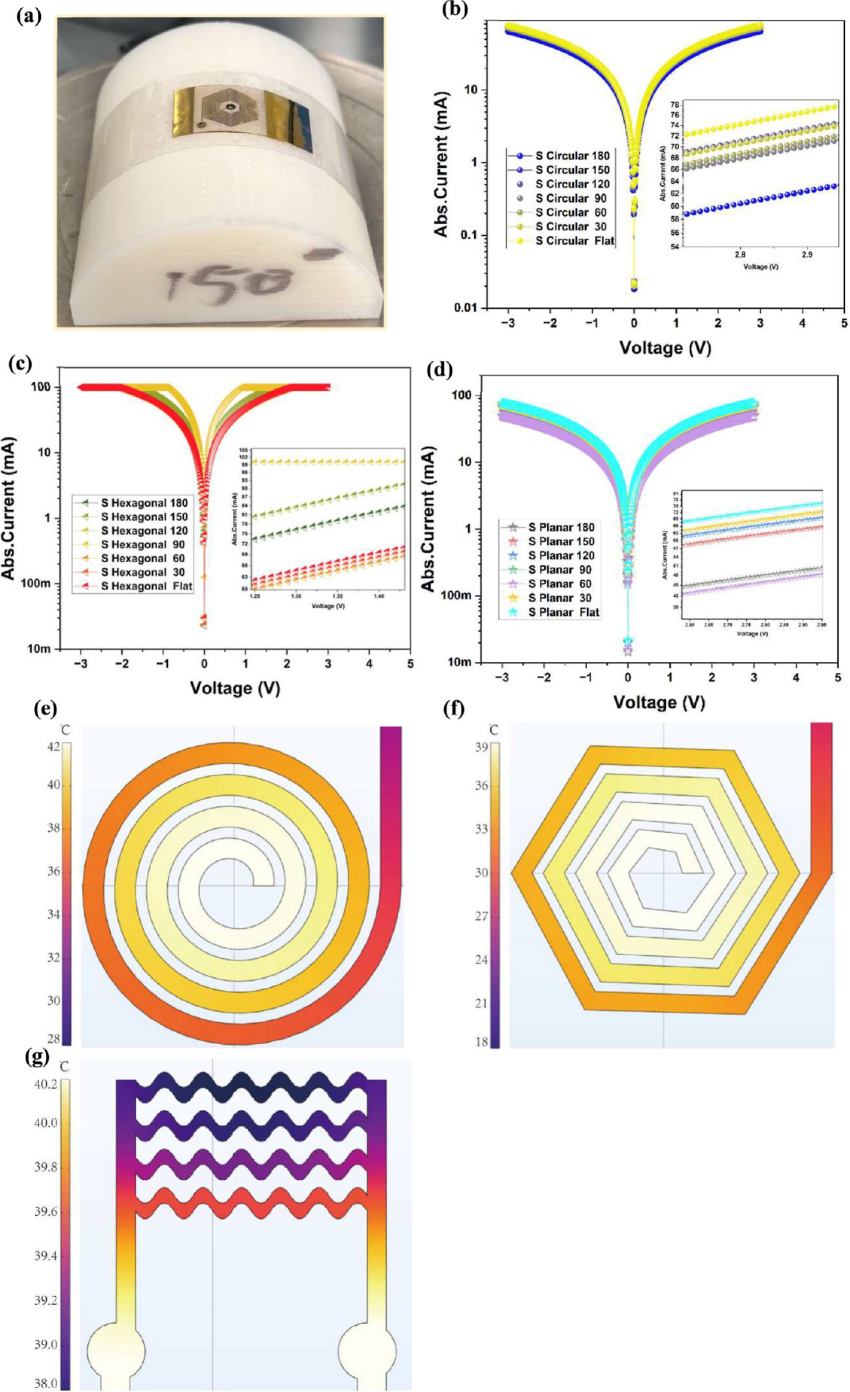
(a) Photograph of the fabricated hexagonal microheater molded on the concave structure of the 3D-printed module used for the bending studies with a bending angle of 150°; (b–d) current vs voltage characteristics of the microheaters circular, hexagonal, and planar design, respectively, with a more detailed magnification of the curves demonstrating the change in the magnitudes of the current with respect to the bending angles varying from 30 to 180°; (e–g) simulation results performed on the different microheater designs including circular, hexagonal, and planar and its different range of the temperatures generated from the microheaters.

The simulation results of the various types of microheaters designed like circular, hexagonal, and planar are presented in Figure 6e–g, respectively. Based on the results, the circular design exhibits the highest temperature (42 °C), followed by the planar (40 °C), while the hexagonal design shows the lowest temperature (39 °C). The circular coils have demonstrated that the slightly higher temperatures might be due to the effective conductive path length and sharp angular edges of the hexagonal pattern (120°) compared to the other two designs, and this further coincides well with the experimental results, i.e., the lower values of the *R*_s_ and conductivity of the circular and planar as compared to the hexagonal. For the circular and hexagonal designs, the heat distribution is more concentrated at the center, whereas the planar design demonstrates less spatial temperature variation. Compared with the experimental results, the simulation results are consistent with the measurement results in the qualitative analysis. Since the simulation results are the temperature of the metal surface under ideal conditions, they should be slightly higher than the reading of the infrared camera, which is also consistent with the situation of a 1 V input.

### 3.6 Biocompatibility Studies (Cell Viability Assay)

Biocompatibility assessments of these types of devices are necessary due to their direct contact with biological tissues and cells. For this purpose, each material that is being used in the fabrication of the microheater can be tested separately to investigate whether each material has any significant impact on a population or a culture of cells. Additionally, two different dyes, such as Acridine Orange (AO) and Propidium Iodide (PI), can be used to distinguish between viable (live) and nonfunctioning (necrotic) cells due to their different excitation and emission values when exposed to different colors of fluorescent dyes. The monomeric binding of acridine orange to DNA results in green fluorescence.^33^ On the other hand, even though propidium iodide is also a DNA-intercalating dye, it is excluded from cells that still maintain their plasma membrane integrity and can only stain cells that have already lost their membrane functions. It is still possible to detect apoptotic cells through PI, but at the same time, PI can stain necrotic cells as well.^34^ This combination of different dyes, therefore, makes them suitable for a viability assay where different colors can be used to distinguish viable cells from others, as depicted in Figure 7. Three images from each well were taken using bright field (BF), Fluorescein isothiocyanate (FITC), and Texas Red (TXRED) channels of the Olympus IX71 microscope. In this instance, Figure 7 demonstrates that each material that is being used in the fabrication of the microheater by itself has no significant reduction in MASMCs viability when compared to a healthy control population. Moreover, the combination of these materials, i.e., the combined microheater itself that contains PAC and gold-patterned PAC, additionally has no significant impact on the viability of MASMCs, which proves its biocompatibility and suitability for in vivo experiments.

**Figure 7:**
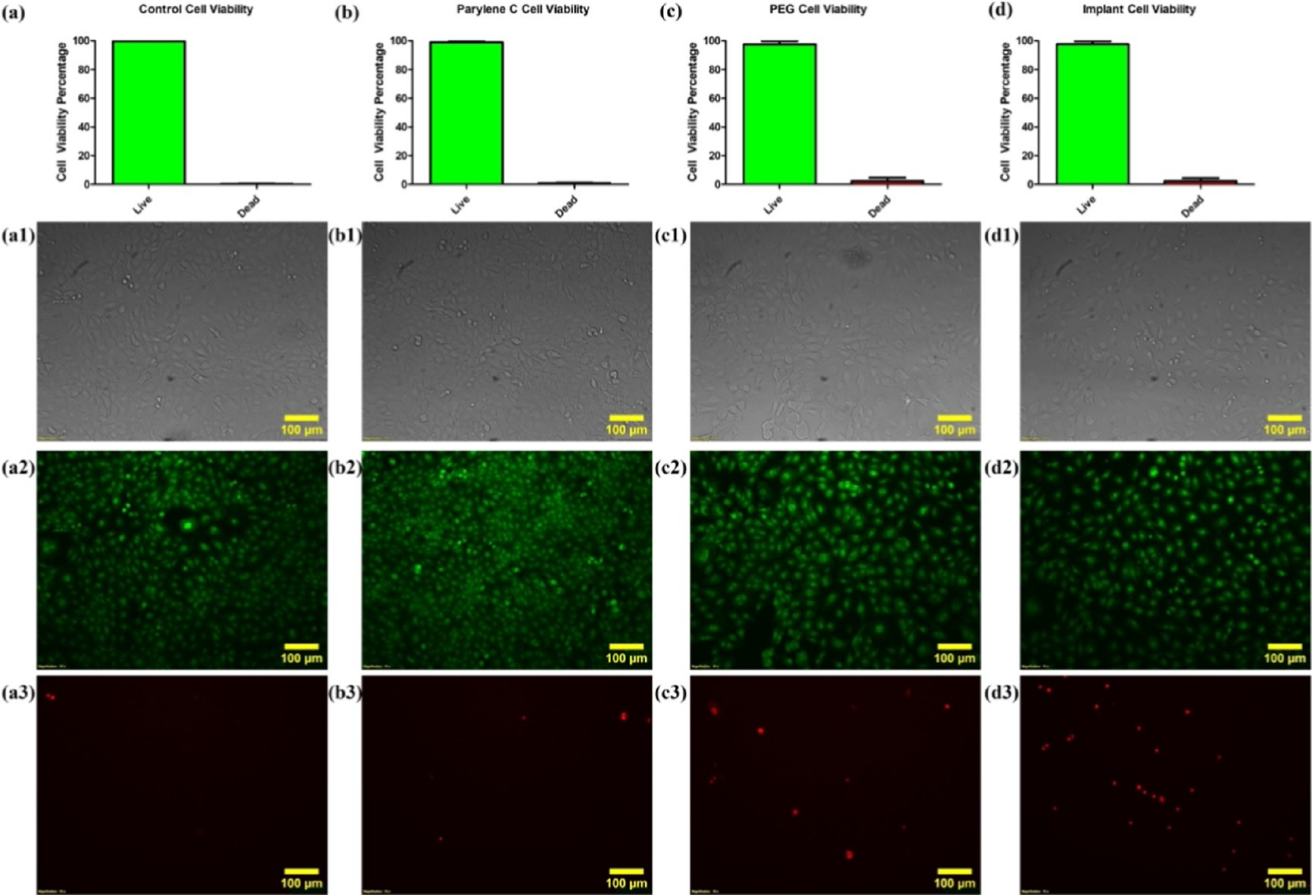
Cell viability results of experimental materials of Parylene C (panel b), gold-patterned Parylene C (panel c), and the patterned microheater (panel d) showing strong cell viability with no significant difference to control experiments (panel a) when compared with one-way ANOVA (p 0.3184, ns, Tukey post-test, *n* = 3). In each group, the second row (a1,b1,c1,d1) represents bright-field images of the well containing its respective material. In contrast, the third row (a2,b2,c2,d2) represents live SMCs stained by acridine orange, and the fourth row (a3,b3,c3,d3) dead/necrotic SMCs stained by propidium iodide.

### 3.7 Reliability Tests of Microheaters for Wearable and Implantable Applications

Mechanical robustness and real-time integration play a vital role in the longer durability of the fabricated microheaters toward wearable and implantable applications. To evaluate the mechanical robustness under multiple bending cycles, changes in the *R*_s_ under various repeated deformations (bend and flat) of 1000 cycles by attaching it to the wooden robotic arm were done for 3 different types of designs (circular, hexagonal, and planar) and are represented in Figure 8a,b. The obtained results suggest that microheaters have shown a maximum deviation of 10%, 19%, and 20% after 1000 cycles for circular, planar, and hexagonal, respectively. These results confirm that the circular designs are very good at stress distributions with much lower variations after repeated use compared to the other two designs. Furthermore, the sharper edges of hexagonal and planar geometries induce a higher stress, leading to unwanted microcracks that induced a drastic variation in *R*_s_ (∼20%) after prolonged bending cycles. Additionally, the microheaters were tested under dynamic and real-time human hand movement by attaching them to various fingers of the hand (index, middle, and thumb). Figure 8c,d shows the picture with the microheater attached to the human hand and the percentage change in the *R*_s_ under 4 different positions and their corresponding pictures. These results show that there is a maximum variation of 28% observed in the *R*_s_ values of different geometries. The deviation in the microheater might be due to the large contact resistance variation at the soldered junctions, along with the irregular surface on the human hand, leading to drastic increase in the change in the resistance values. However, these variations can be easily nullified by using more robust and advanced wire bonding techniques at lower temperatures and using wireless data transfer techniques, eliminating the need for the extra-long wires and junction soldering points. Next, a soft elastomeric PDMS-based artificial human hand with fingers was prepared and used for the wearable thermotherapy measurements to fully biomimic a human hand. Here, PDMS is chosen for physical emulation of the human hand owing to its tunable Young’s modulus of elasticity and thermal conductivity that match the human skin and provide a good platform for the heat dissipation and uniform spread of the thermal profiles. The electrothermal measurements were performed by attaching the microheaters to the biomimicked human hand, as shown in Figure 8e,f. The results demonstrate that variation in the temperature is minor for all geometries and sizes in comparison with the normal rigid surface. Three different temperature ranges (low, medium, and high) were selected for the detailed comparison of the performance, and all the results show that the temperatures are slightly higher without the hand owing to the absorption of a minor (<5%) amount of heat into the elastomeric hand, resulting in an overall reduction in the temperatures. Finally, these results demonstrate that our laser-patterned Au-based microheaters are mechanically robust and maintain a good, stable behavior under various bending cycles along with real-time wearable or joint-mounted applications.

**Figure 8:**
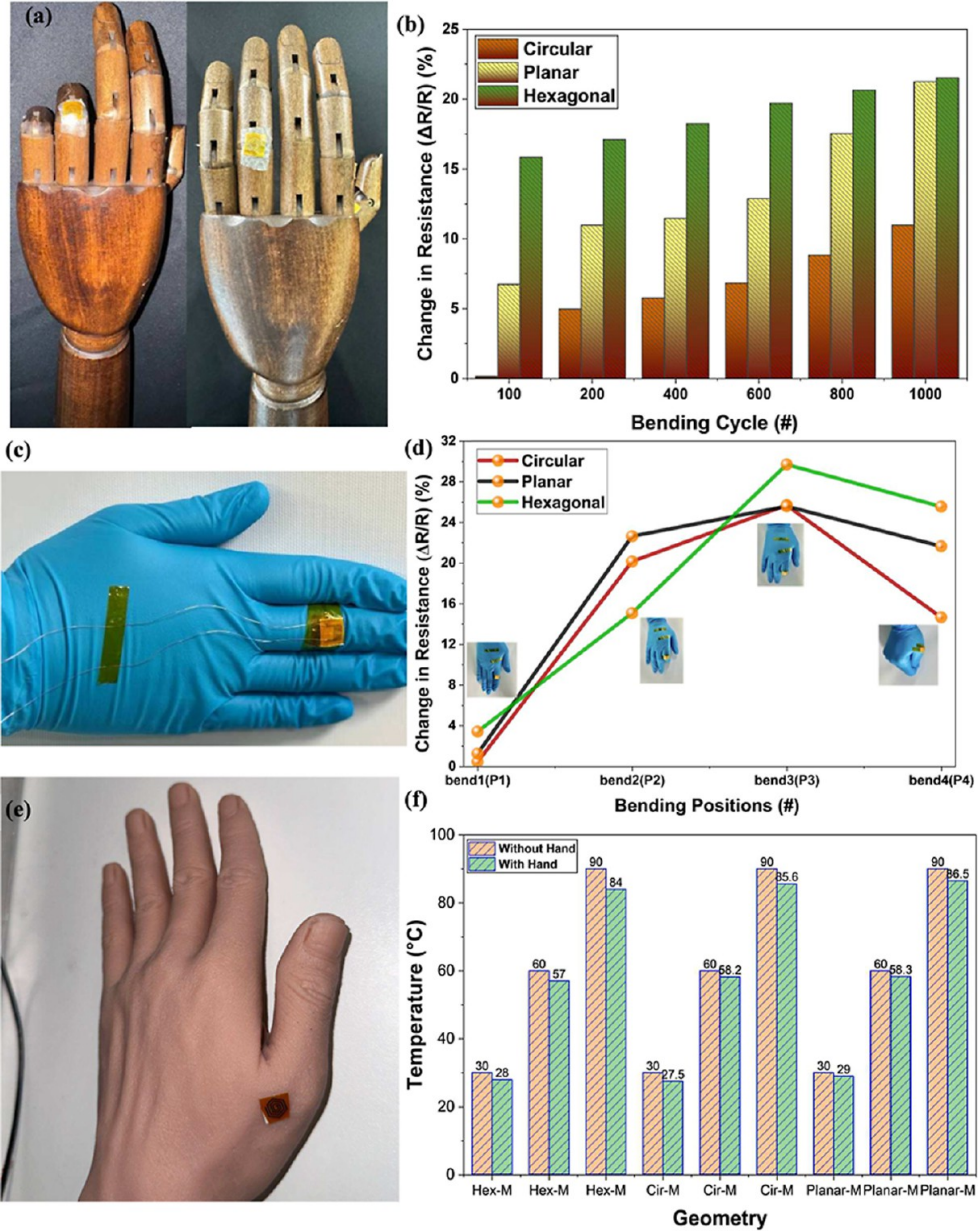
Real-time dynamic bending tests on the microheaters: (a,b) picture showing the microheater attached to the robotic hand and performing repeated bending cycles (100–1000) and the corresponding performance variation in the *R*_s_ for different geometries and sizes; (c,d) a real-time picture of the microheater attached to the human hand showing the dynamic hand movements in various positions and its change in *R*_s_ corresponding to each of the bending portions; (e,f) similarly, a biomimic of the human skin using an elastomeric hand showing similar shape and the corresponding thermal response on static and human hand-like surfaces for different geometries and its corresponding sizes.

### 3.8 Soaking Tests of Microheaters in PBS

To further analyze the reliability of the laser-fabricated microheaters, soaking tests were performed for various time intervals (24, 48, and 72 h) in phosphate buffer solution (PBS) at 37 °C as shown in Figure 9a. Followed by this, the *R*_s_ measurements were performed on the soaked gold film-based heaters for all the fabricated designs, including circular, hexagonal, and planar, as shown in Figure 9b. The results demonstrate that a minor variation in the change in the *R*_s_ (<5%) occurred after the soaking tests were performed, and the primary reason for such a meagre variation in *R*_s_ is mainly due to the full encapsulation using PAC around the coils. The encapsulation layer is strongly adhered to the substrate, providing a hydrophobic and defect-free/pinhole-free effective barrier on top and protecting against moisture, humidity, and corrosion. It is well-known that PAC is well-reported as an encapsulation layer for various implantable and medical applications, owing to its excellent biocompatibility, chemical inertness, and low permeability. Further, no visual degradation or surface compositional change was observed, clearly confirming that proper encapsulation was done to avoid any metal ion leaching. Finally, the pH value of the solution was measured before and after soaking tests to find any traces of gold metal ions diffused into the PBS solution, ensuring the suitability of the microheaters for wearable and implantable applications. A detailed comparison table with state-of-the-art fabricated heaters was provided that includes different fabrication approaches, such as inkjet printing, 3D printing, photolithography, and laser, as shown in Table 1. Different fabrication strategies, such as photolithography, printing technologies, spin coaters, spray coaters, etc., were explored for the fabrication of different types of heaters for a wide variety of applications, including sensors, wearables, and implantable devices. However, the state-of-the-art photolithographic process needs sophisticated facilities that are quite expensive, require a lot of sophisticated equipment and skilled labor, and are time-consuming as well. On the other hand, highly conducting ink preparation is complex and still needs a lot of optimizations with the desired viscosity of the ink, stability and reliability of the inks, adhesion issues with the substrates, poor device-to-device repeatability, performance variation in the devices due to environmental factors such as humidity, and moisture improvements. On the other hand, advanced picosecond lasers offer a unique fabrication strategy, offering flexibility with respect to the target materials, opportunity for various complex designs and geometries, and rapid processing speeds supporting mass manufacturability. This type of fabrication approach lays the foundations for the fabrication of various studies, like surface nanostructuring, synthesis of nanomaterials, post-treatment of nanomaterials, etching of the layers, drilling of through-glass vias (TGV), etc.

**Figure 9:**
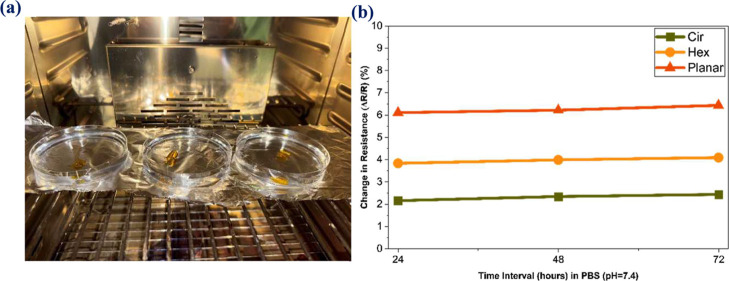
Soaking tests in PBS solution: (a) photographic picture of three design samples showing the dissolution tests performed in an oven at 37 °C, and (b) percentage change in *R*_s_ after 24, 48, and 72 h, respectively, and all the samples have shown minor variations in the sheet resistance after 3 days.

**Table 1:** A Detailed Comparison Table Showing Various State-of-the-Art Microheaters Demonstrated Using Various Fabrication Strategies

fabrication method	process complexity	material	design flexibility	substrate compatibility	min. feature size (μm)	maximum *T* (°C)	cost	ref
photolithography	high (5–6 steps)	PI/Ti/Au	poor	moderate	5 μm	70	very high	^35^
3D printing	less	paper/glass	good	good	1–5 mm	60	low	^36^
inkjet printing	less	PET/Ag	good	good	60 μm	100	low	^37^
spray coating	moderate	glass/SnO_2_	poor	good	>100 μm	150	low	^38^
3D printing + laser	moderate	PI/Cu	good	good	60 μm	150	moderate	^39^
picosecond laser (UV)	less	PI/Ti/Au	very good	very good	20–30 μm	100–110	low	this work

## 4 Conclusions

In conclusion, we have successfully proposed and demonstrated a unique fabrication approach using an advanced ablation picosecond laser for the precise patterning of the ultrathin metallic films of any desired thickness, typically ranging from nanometers to micrometers, on conformable, transparent, and sensitive emerging thin layers of a polymer substrate such as PAC and polyimide. This type of fabrication methodology using advanced lasers is unique and the least explored for the fabrication of microheater applications. This laser-based method offers numerous advantages in terms of accuracy, precision, design flexibility, rapid prototyping, material versatility, and a contact-free approach. More importantly, the versatile approach using lasers operates in a cleanroom-free environment and at room temperature, offering cost effectiveness, environmental stability, scalability, and minimal thermal damage to provide the best possible solution for small- to medium-scale manufacturability. Various designs, including circular, hexagonal, and planar patterns, were successfully fabricated by using the approach and were demonstrated for microheater applications. The microheaters are tailored for the targeted temperature ranges varying from 30 to 100 °C for the dedicated wearable thermal therapy applications such as accelerated wound healing. The fabricated microheater designs in small to extra-large sizes have successfully demonstrated good uniform heat deposition, superior mechanical flexibility, and faster response and recovery times. Among all, the circular has demonstrated excellent power efficiency with a very good uniform radial distribution of heat in a very short period of less than 30 s, particularly suitable for the targeted wearable applications. Further, electrical, electrothermal, and mechanical flexibility characterizations demonstrated that the *R*_s_, conductivity, and thermal response vary with the design and size of the microheaters and thermal response, and response time varied with the structure and size of the microheaters. Mechanical bending experiments reveal excellent mechanical robustness and suggest that the microheaters are reliable for long-term use on various nonuniform surfaces like the human body. Next, the biocompatibility studies reveal the excellent compatibility of the PAC and the gold electrodes for wearable and implantable biomedical applications. The proposed work helps in the advancement of the field of flexible electronics and addresses important challenges in medical devices, especially for wearable smart bandages for wound healing applications. Additionally, the laser-based fabrication technique offers a unique advantage in terms of room temperature etching without affecting the inherent properties of the materials and eliminates the need for unwanted toxic chemicals, offering a cleanroom-free methodology, mainly overcoming the technical drawbacks of the current state of microfabrication techniques. Finally, the proposed research paves the pathway for a scalable, small-scale manufacturable and energy-efficient method for the next generation of wearable and implantable biomedical devices toward innovative thermal therapy applications.

## Supplementary Material



## Notes

Laser power optimization studies, microscopic images of the fabricated microheaters, XRD and FTIR characterizations, optimization parameters of the picosecond lasers used for the fabrication of different designs of the microheater, high- and low-magnification images of the *I*–*V* characteristic measurements under bending studies, experimental setup showing pulse measurements, flowchart and its control algorithmic flow demonstrating the feedback mechanism, plots showing the mechanical bending studies, and electrochemical impedance spectrum (PDF)
