# Supplementary material for: Lab to Fab Process Using Ablation Lasers: A Lightweight, Flexible, and Biocompatible Microheater for Wearable Therapy Applications
Source: ACS Appl Bio Mater. 2025 Oct 17;8(11):9931–46. doi: 10.1021/acsabm.5c01263 (PMC12628293; doi:10.1021/acsabm.5c01263)
Supplement: Supplementary file 1 [file mt5c01263_si_001.pdf]

Supporting Information
on

Lab to fab process using ablation lasers: A light weight, flexible and biocompatible Microheaters for wearable therapy applications

Bhavani Prasad Yalagala[1], Tahereh Masalehdan[1], Changhao Ge[1], Mahmut Talha Kirimi[3], John Mercer[3], Morteza Amjadi Kolour[2] and Hadi Heidari[1]*

[1]Microelectronics Lab (meLAB), School of Engineering, University of Glasgow, G12 8QQ, UK

[2] School of Biomedical Engineering, University of Glasgow, G12 8QQ, UK.

[3]Institute of Cardiovascular and Medical Sciences/British Heart Foundation, University of Glasgow; UK

*Corresponding Author: hadi.heidari@glasgow.ac.uk

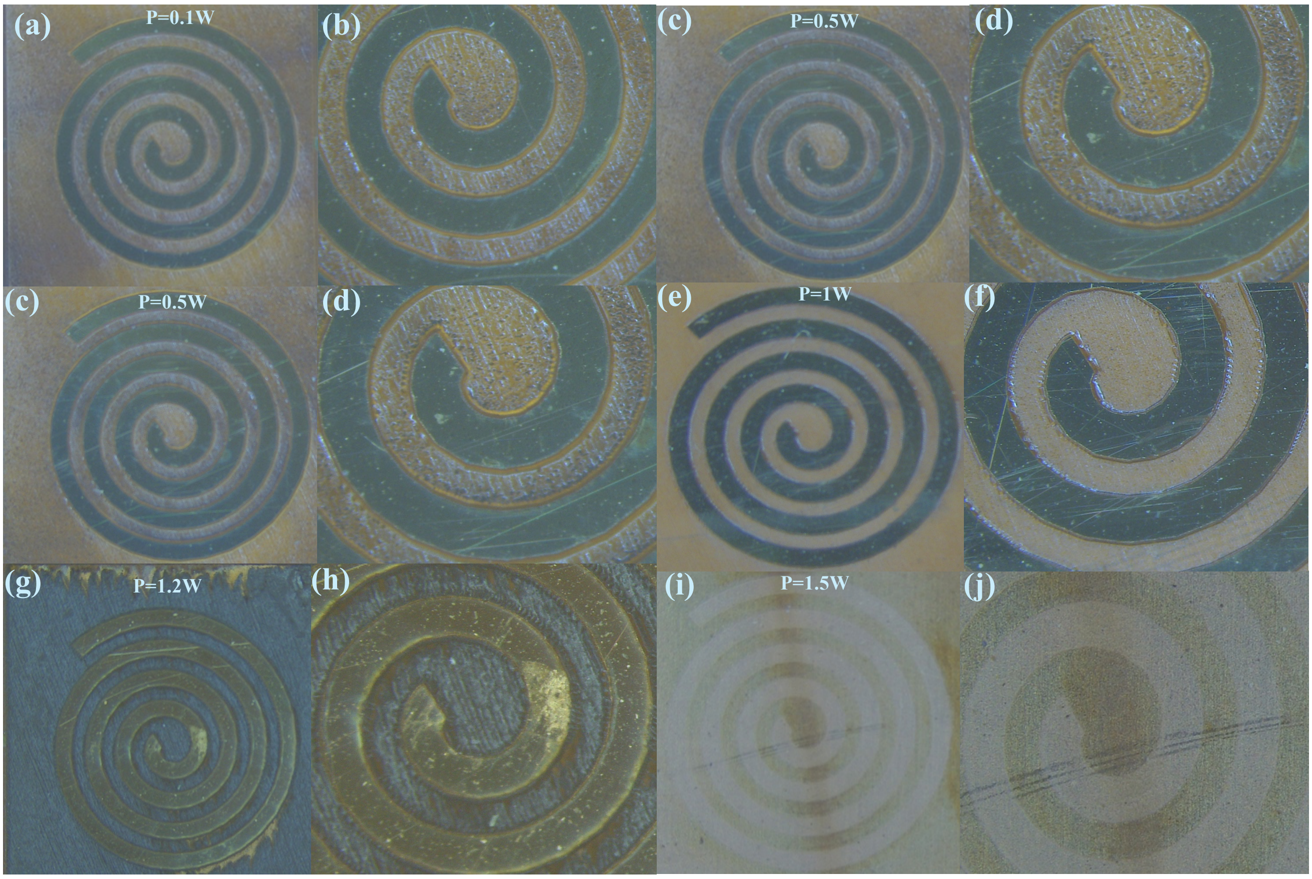

Figure S1: Laser power optimisation studies (a)-(j) low and high magnification images of the circular images for a lower to a higher power in the increasing order clearly showing that at a low power values there is still residues present after the hatching of the gold layer conversely the higher power has more burns in the hatch region clearly showing the change in the colour of the intermediate regions.

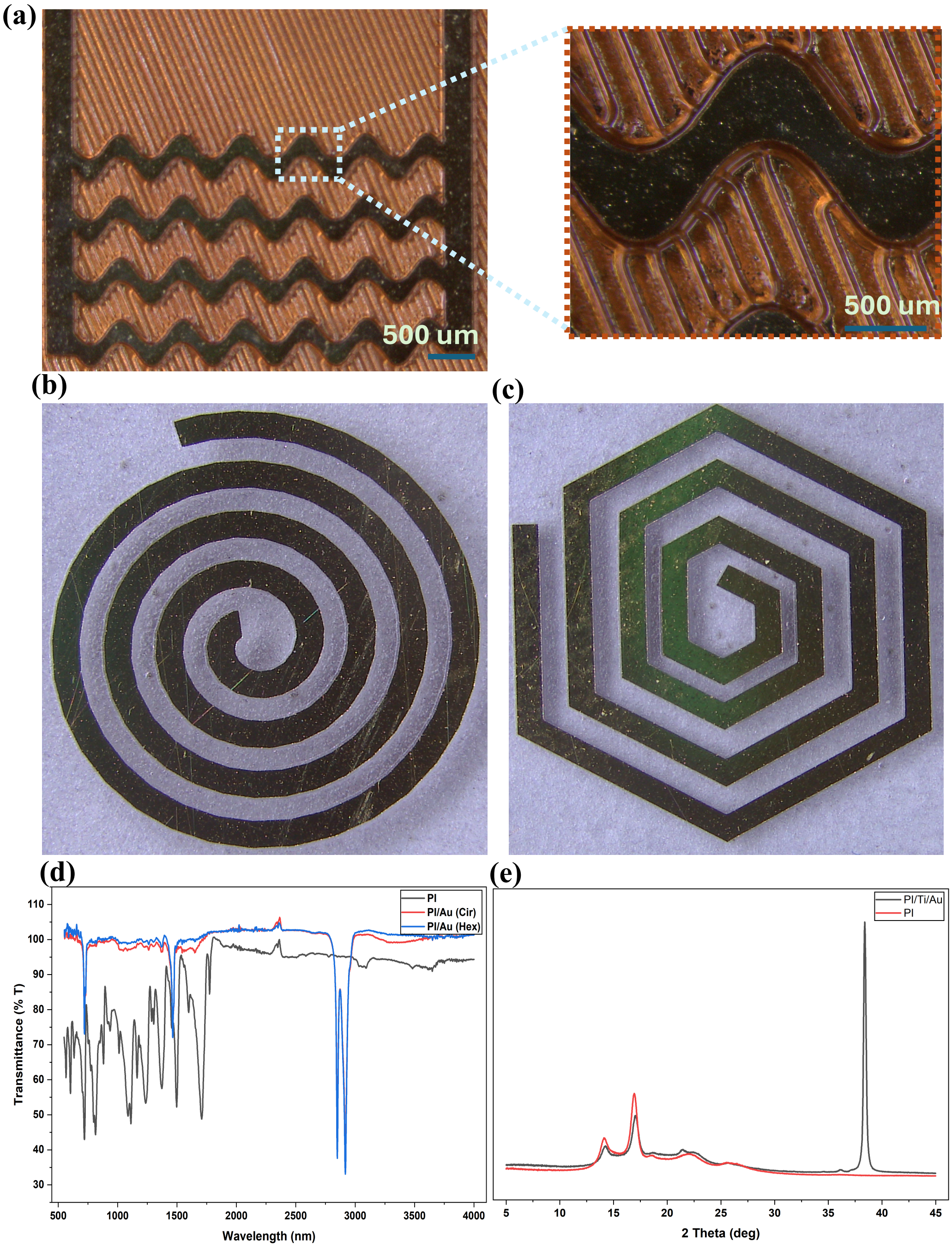

Figure S2: (a)-(c) Microscopic images of the fabricated microheaters showing no degradation on the surface after the picosecond laser ablation on Au electrodes XRD and (d) and (e) FTIR and XRD on the bare PI (before laser treatment) and after laser treated/etched showing no signs of the graphene/carbon peaks confirming on adverse effects on the substrate.

Table S1: Optimisation parameters of the picosecond lasers used for the fabrication of different designs of the microheater

S.No	Design	Power (mW)	Frequency (kHz)	Heat copy (mW)	Scanning speed (mm/s)	Number of repetitions
1	Circular	1000	50	200	120	2
2	Hexagonal	1000	50	200	120	2
3	Planar	1000	50	200	120	2
4	Lowest values	100	25	100	75	1
5	Highest values	1500	75	400	180	4

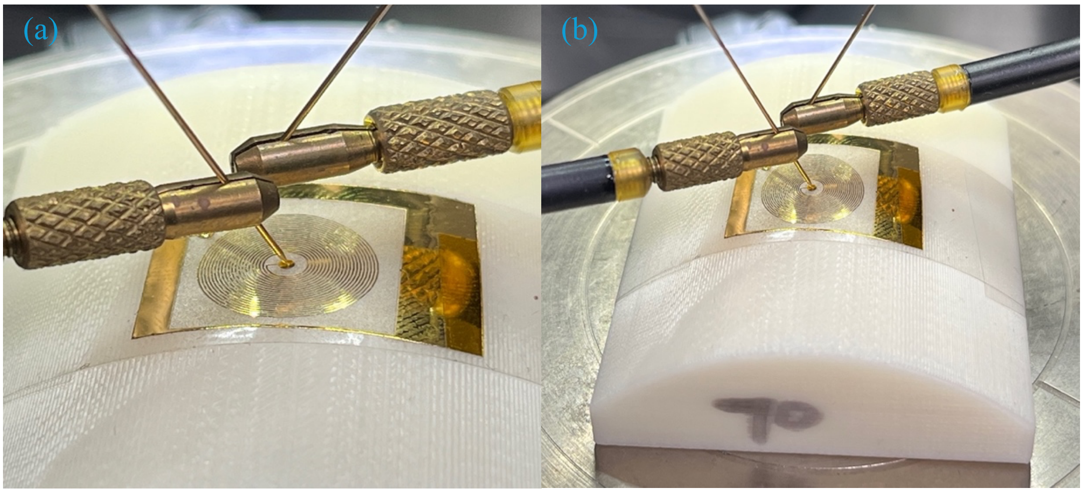

Figure S3: (a) and (b) high and low magnification images of the I-V characteristic measurements of the circular microheaters demonstrating the mechanical flexibility under the bending angle of 70° on a 3D printed module.

(a)

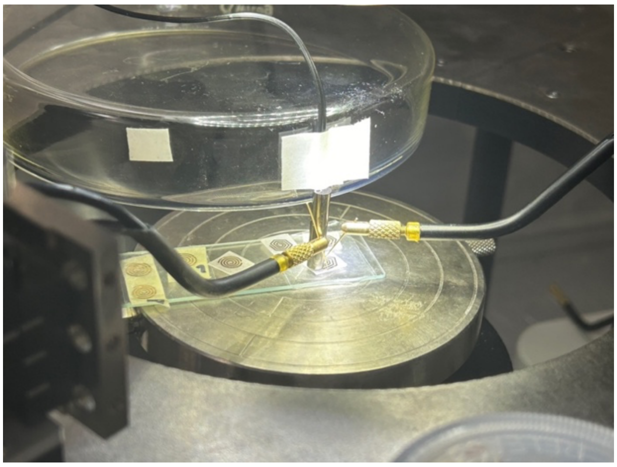

(b)

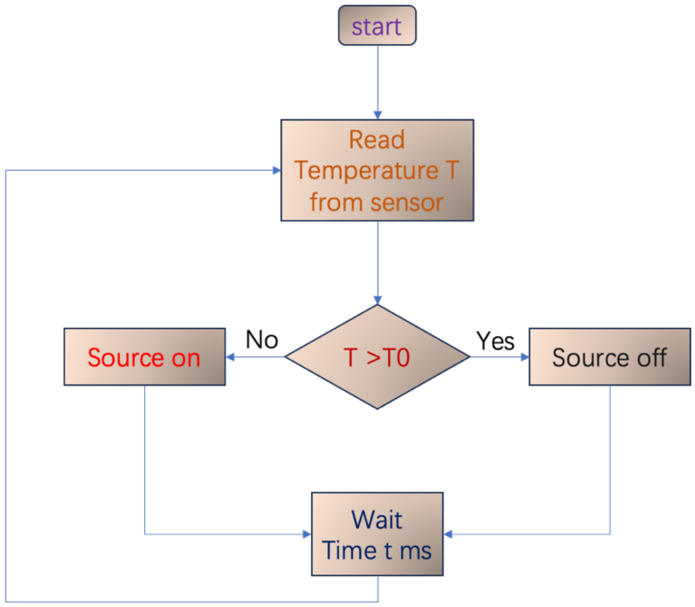

Figure S4: (a) Experimental setup showing the pulse measurements performed using the commercial thermal sensor integrated with the microcontroller to measure the response and recovery time of the microheaters, and (b) the proposed flowchart and its control algorithmic flow demonstrating the feedback mechanism used to control the response from the microheaters by regulating the input power to the microheater.

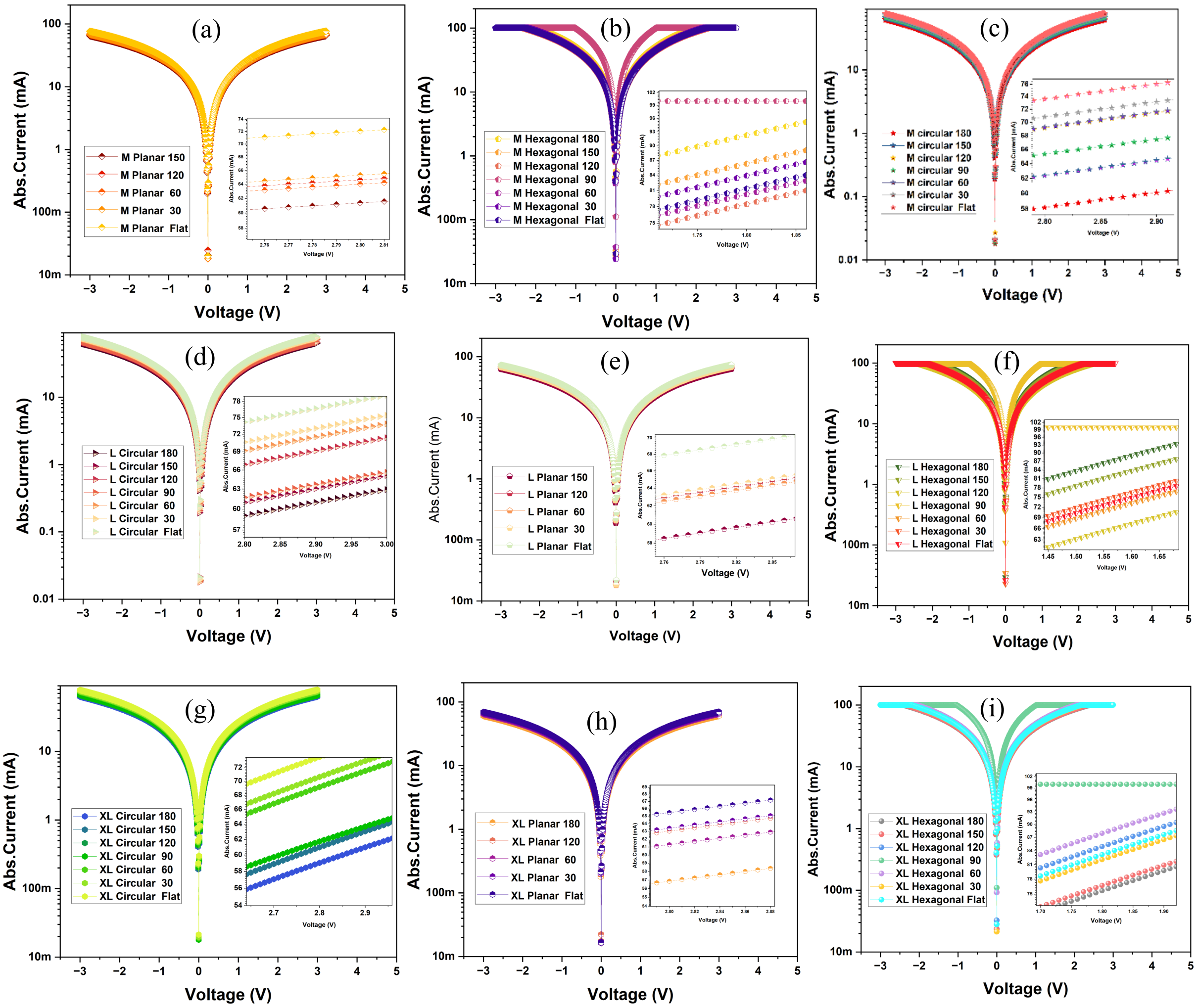

Figure S5: (a)-(i) Plots showing the mechanical bending studies performed on the Parylene C based flexible microheaters using the I-V measurements on various designs and multiple sizes like medium, large and extra-large under different bending angles varying from 0-180°.

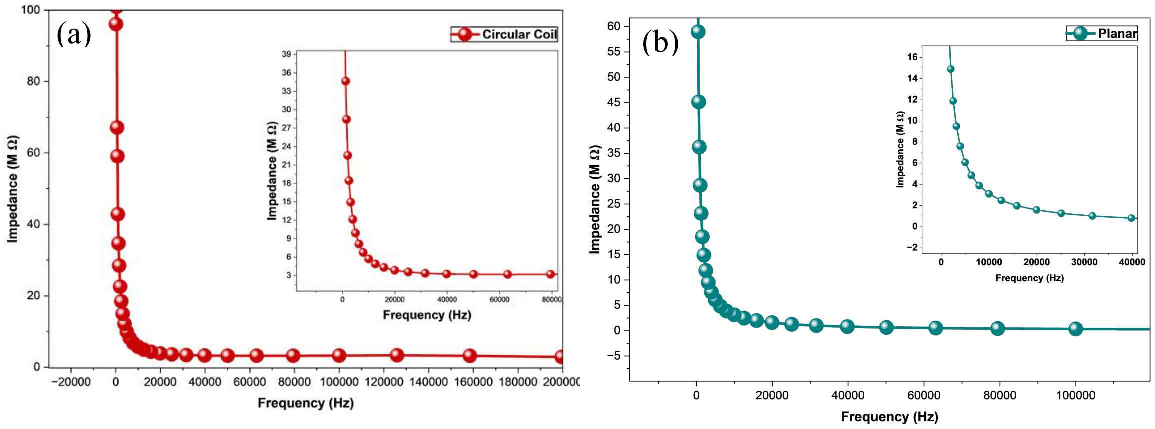

Figure S6: Electrochemical impedance spectrum of (a) Circular microheater and (b) planar microheater and inset showing its magnified images in the low frequencies.

Table S2a: Heating Efficiency of the "Circular" based microheaters

T (°C)	XL	L	M	S
30	27.47253	29.60156	39.5132	54.53752
40	22.30276	29.37893	38.82892	49.63272
50	23.73042	29.46781	38.13883	51.72771
60	23.60383	30.31474	37.56292	49.666
70	25.36783	31.47425	38.4574	51.3347
80	25.63555	32.6833	40.98361	50.23106
90	27.7431	34.25898	43.92157	51.23701
100	28.06771	35.73343	46.39107	51.28205

Table S2b: Heating Efficiency of the "Hexagonal" based microheaters

T (°C)	XL	L	M	S
30	95.12937595	39.37008	35.61254	65.35948
40	66.72894702	37.89817	41.02564	57.14286
50	67.8119349	39.49967	37.93915	58.64415
60	67.20430108	39.22722	40.52685	64.83823
70	67.06908115	39.55321	40.3825	64.71989
80	62.12530674	38.90849	43.69674	58.96806
90	62.5	35.71829	39.80009	60.53269
100	-	37.34391	40.49751	64.50989

Table S2c: Heating Efficiency of the "Planar" based microheaters

T (°C)	XL	L	M	S
30	55.55555556	47.61905	46.51163	61.7284
40	43.52367688	45.95166	49.54788	61.7284
50	44.94382022	47.78211	57.54844	56.98655
60	48.48484848	50.0	66.02621	51.26452
70	51.94805195	53.50054	71.83908	61.3384
80	56.85964197	58.50804	79.15358	66.13757
90	60.37606558	62.42197	82.41402	73.97909
100	64.77732794	63.01843	88.41538	79.40109
